# Solid–Liquid–Solution Phases in Poly(diallyldimethylammonium)/Poly(acrylic acid) Polyelectrolyte Complexes at Varying Temperatures

**DOI:** 10.1021/acs.macromol.4c00258

**Published:** 2024-02-22

**Authors:** Chikaodinaka I. Eneh, Kevin Nixon, Suvesh Manoj Lalwani, Maria Sammalkorpi, Piotr Batys, Jodie L. Lutkenhaus

**Affiliations:** † Artie McFerrin Department of Chemical Engineering, Texas A&M University, College Station, Texas 77843, United States; ‡ Department of Chemistry and Materials Science, Aalto University, P.O. Box 16100, Aalto 00076, Finland; § Department of Bioproducts and Biosystems, Aalto University, P.O. Box 16100, Aalto 00076, Finland; ∥ Academy of Finland Center of Excellence in Life-Inspired Hybrid Materials (LIBER), Aalto University, P.O. Box 16100, Aalto 00076, Finland; ⊥ Jerzy Haber Institute of Catalysis and Surface Chemistry, Polish Academy of Sciences, Niezapominajek 8, Krakow 30-239, Poland; # Department of Materials Science and Engineering, Texas A&M University, College Station, Texas 77840, United States

## Abstract

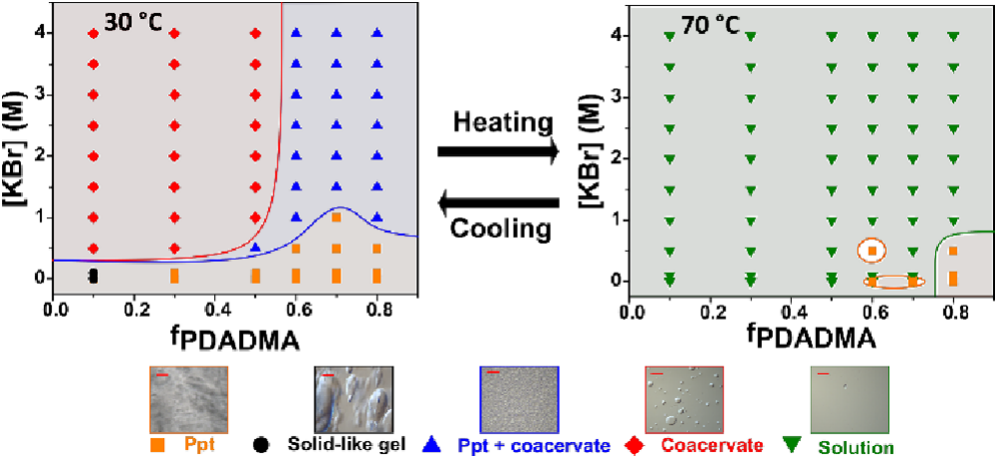

The coacervation and complexation of oppositely charged polyelectrolytes are dependent on numerous environmental and preparatory factors, but temperature is often overlooked. Temperature effects remain unclear because the temperature dependence of both the dielectric constant and polymer–solvent interaction parameter can yield lower and/or upper critical solution phase behaviors for PECs. Further, secondary interactions, such as hydrogen bonding, can affect the temperature response of a PEC. That is, mixtures of oppositely charged polyelectrolytes can exhibit phase separation upon lowering and/or increasing the mixture’s temperature. Here, the phase behavior of poly(diallylmethylammonium)/poly(acrylic acid) (PDADMA/PAA) complexes under varying KBr ionic strengths, mixing ratios, and temperatures at a fixed pH (in which PAA hydrogen bonding can occur) is examined. At room temperature, the PDADMA/PAA PECs exhibit four different phase states: precipitate, coexisting precipitate and coacervate, solid-like gel, and coacervate. Variable-temperature optical microscopy reveals the upper critical solution temperature (UCST) at which each phase transitioned to a solution state. Interestingly, the UCST value is highly dependent on the original phase of the PEC, in which solid-like precipitates exhibit higher UCST values. Large-scale all-atom molecular dynamics (MD) simulations support that precipitates exhibit kinetic trapping, which may contribute to the higher UCST values observed in the experiment. Taken together, this study highlights the significance of temperature on the phase behavior of PECs, which may play a larger role in stimuli-responsive materials, membraneless organelles, and separations applications.

## Introduction

Phase separation is entropically favored when two polyelectrolytes (PEs) of opposite charge are mixed, forming intrinsic PE–PE ion pairs by the release of small counterions^1−3^ and reorganization of solvent structure.^4^ For instance, while the entropic contributions have been popularly attributed to counterion release, a recent study has shown that the primary entropy contribution in weak to intermediate electrostatic strengths comes from the temperature dependence of dielectric constant of water and solvent reorganization.^4^ This process results in the formation of a polymer-poor phase called the supernatant and a polymer-rich phase called a polyelectrolyte complex (PEC). The polymer-rich phase may exist as a solid-like precipitate or a liquid coacervate. Factors such as the ionic strength, pH, dielectric constant, polymer solution concentration, and linear charge density can influence the phase separation behavior by influencing the strength of the PE–PE electrostatic interactions.^2,5−8^ However, the effects of temperature on the phase behavior of PECs are not well-documented and existing studies have shown conflicting results.^9−12^

Polyelectrolyte complex phase boundaries have been explored with respect to pH, mixing ratio, salt type, and concentration.^6,13−15^ An early theory defining the coacervate/solution boundary is the Voorn–Overbeek (VO) theory, which applied both Flory–Huggins (FH) theory of mixing for polymer solutions and Debye–Hückel (DH) theory of dilute electrolytes.^16,17^ Since then, more theoretical descriptions^18−23^ and experimental approaches, such as UV–vis spectroscopy,^5^ thermogravimetric analysis (TGA),^24^ optical microscopy,^6,25^ and ionic conductivity,^26^ have explored the coacervate/solution boundary. However, the coacervate/precipitate boundary, which distinguishes between solid and liquid phases, has so far received less attention^26−28^ and is not adequately described by existing theory.

Polymer assembly phases and phase separation can readily be described by field theory approaches.^29^ Although limited in length and time scales to molecular-level description and localized assembly, particle-based and molecular simulation methods provide additional insight into PE phase behavior.^30,31^ Atomistic-level molecular dynamics (MD) simulations have been utilized to chart the origins of a number of functional group-level dependencies in phase separation, such as investigating the shift in PE p*K*_a_,^32,33^ extracting the governing molecular interaction mechanisms behind the phase separation,^34,35^ but also in examining the local structural changes of the complexes.^36^ Less limited in their description time and length scale, but also lacking the atomistic detail resolution, coarse-grained molecular modeling approaches have provided access to PE dynamics in the assemblies^37^ and their rheological properties.^38^ In contrast to atomistic simulations, coarse-graining allows PE assemblies to achieve equilibrated states, which has been used to study, e.g., PE partitioning between coacervate and supernatant phases, and also the interfacial properties of the two phases.^39^

The emergence of coacervate and solution phases with regard to temperature depends on competing factors such as electrostatic interactions, hydrogen bonding, and hydrophobic interactions. VO theory includes a temperature dependence through the FH parameter (χ), the solvent dielectric constant (ε), the Bjerrum length (*l*_B_), and the Debye screening length (κ^–1^).^9,17,40^ This temperature dependence leads to two possibilities for the coacervate/solution system: lower critical solution temperature (LCST) and upper critical solution temperature (UCST) phase behaviors. For example, LCST behavior leads to phase separation with an increase in temperature and can be attributed to a decrease in the dielectric constant, an increase in the dipole–dipole interaction energy, and an increase in the polymer–solvent interaction parameter. On the other hand, UCST behavior leads to phase separation with decreasing temperature, which can be attributed to the polymer–solvent interaction parameter decreasing with increasing temperature.^9^

PECs of poly(diallyldimethylammonium) and poly(acrylic acid) (PDADMA and PAA, respectively) are interesting for their potential applications in drug delivery systems, electrochemical devices, and self-healing materials.^10,41−44^ PDADMA is a strong PE that is fully ionized over a wide range of pH values, and PAA is a weak PE that can be partially or fully ionized, depending on the pH. For example, at pH > 10, PAA homopolymer films are fully ionized, but at pH < 2, PAA films are fully protonated.^44^ Studies on the phase behavior of PDADMA/PAA PECs have shown that—depending on the salt, ionic strength, pH, and mixing ratio—a white solid precipitate, coacervate, or a combination of both phases is obtained.^27,28,45^ For example, PDADMA/PAA complexes at pH 10 exhibited transitions from precipitate to a mixed precipitate + coacervate phase with increasing PAA content; however, further increase in the PAA content yielded mixed phases.^28,45^ Elsewhere, Salehi et al. demonstrated that PDADMA had relatively weak electrostatic interactions with PAA when compared to other selected polycations.^27^ Steric hindrance from the methyl groups on the quaternary amine groups of the PDADMA chains may have contributed to this weak interaction. The strongest interactions for PDADMA/PAA complexes were observed at pH 3, where the observed critical salt concentration was about five times that at pH 7. Contrary to expectations based on the relative charge densities of PDADMA and PAA at pH 3, precipitation rather than coacervation occurred, and exponential growth was observed for multilayer films in the absence of salt. These results agree with a shift in the p*K*_a_ of PAA, which contributed to enhanced electrostatic interactions as well as some contributions from secondary hydrogen bonding interactions at pH 3.

Interestingly, both LCST and UCST behavior have been reported for different polyelectrolyte complex systems. For example, the UCST behavior in PDADMA/PAA complexes in acidic media was studied using light scattering.^10^ The effect of temperature on the second virial coefficient, A_2_, of PDADMA/PAA complexes in 0.1 M HCl was monitored as a measure of solvent quality, which relates to phase separation. Elsewhere, Wang and Schlenoff^25^ demonstrated the coalescence of PDADMA/polystyrenesulfonate (PSS) coacervate droplets with increasing temperature using optical microscopy, suggesting similar UCST behavior. In contrast, Ali et al. prepared PDADMA/PSS PECs at a higher polymer concentration and obtained LCST behavior as the PEC solution went from clear to cloudy upon heating, suggesting LCST behavior.^11^ This LCST behavior was described using a coarse grain model and theoretical models that captured the influence of temperature on the dielectric constant and the resulting phase separation.^9,46^ Bringing these two results together, Ye et al. recently observed both UCST and LCST behavior for PDADMA/PSS PECs at low and high polymer concentrations, respectively. Both UCST and LCST behaviors have been observed in other similar biological polymeric materials such as polyampholytes, proteins, and intrinsically disordered proteins.^12,47−49^

Taken together, the preceding literature suggests that whether a PEC undergoes an LCST or an UCST may be influenced by the presence of hydrogen bonding interactions in one or both of the components in which hydrogen bonding interactions promote UCST-type behavior. We hypothesize here that purposefully harnessing hydrogen bonding interactions in PDADMA/PAA complexes can promote UCST behavior, as well as solid precipitate formation under certain conditions. We also hypothesize that precipitates would exhibit significantly different UCST behavior relative to coacervates due to the different natures of the respective solid and liquid phases.

In this paper, we reveal how temperature affects the coacervate/precipitate boundary and, in turn, the temperature-induced disassembly of the PDADMA/PAA complex. We chose this system because it had been shown previously^27^ to exhibit solid, liquid, and solution phases at acidic pH values at room temperature. PDADMA/PAA PECs are prepared at pH 3.22 for varying PDADMA:PAA mixing ratios and KBr concentrations. We distinguish solid–liquid and liquid–liquid phase separations using UV–vis spectroscopy and optical microscopy at varying temperatures, resulting in a series of phase maps that display UCST behavior. Large-scale MD simulations were implemented to understand the PE interactions and changes in dynamics in the molecular level rearrangement as well as possible differences between the solid and liquid phases. We also explored the coacervate/solution boundary using thermogravimetric analysis (TGA) of both dense and dilute phases. Put together, this work shows how the temperature and hydrogen bonding can influence the original phase (whether solid precipitate or liquid coacervate) of PECs, leading to different UCST values.

## Experimental Section

### Materials

Poly(diallyldimethylammonium) (PDADMA, *Mw* = 200,000–350,000 g/mol, 20 wt % solution) and poly(acrylic acid) (PAA, *Mw* = 100,000 g/mol, 35 wt % solution) were purchased from Sigma-Aldrich. Potassium bromide (KBr) was purchased from Alfa Aesar.

### Preparation of Solutions

PDADMA and PAA solutions were diluted, and placed in individual 15 kDa molecular weight cut-off dialysis bags, and dialyzed against pure Milli-Q water for 2 days. The dialysis water was changed intermittently, and dialysis was concluded when the conductivity of the dialysis water was back down to ∼2 μS/ms. The dialyzed polyelectrolyte solutions were then placed in 50 mL centrifuge tubes and lyophilized for 2 days to obtain a dry powder. Stock solutions of 0.5 M by repeat unit of both PDADMA and PAA were prepared and pH-adjusted to a value of 3. A KBr stock solution was prepared at 4 M (pH 5.5).

### Polyelectrolyte Complexation

**Scheme 1:**
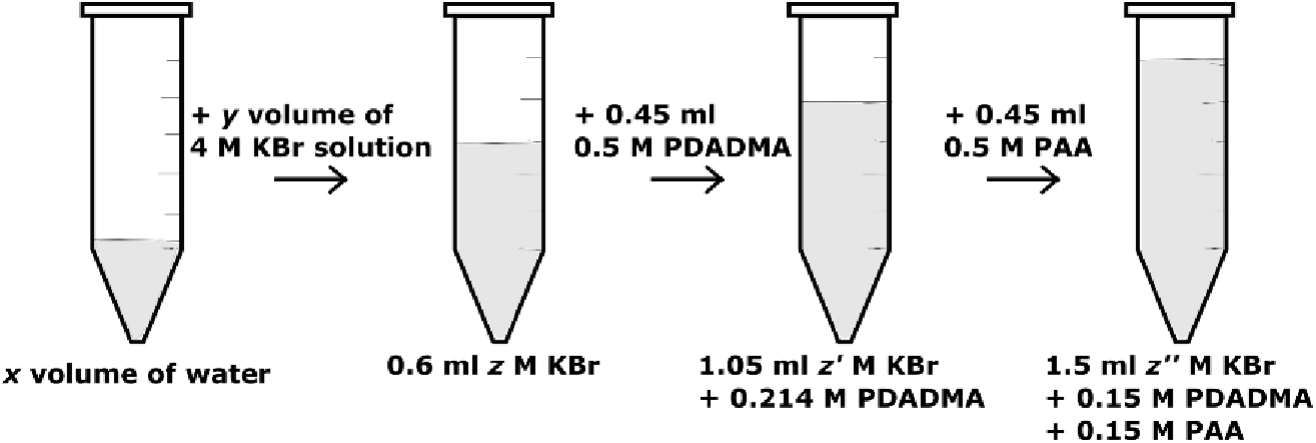
Polyelectrolyte Complexation Procedure Showing Component Volumes and Concentrations for a 0.5 Mole Fraction PDADMA Complex at *z*” M KBr

All PECs were prepared following the same mixing protocol to eliminate any differences in properties as a result of changes in the kinetic pathway.^50,51^ Immediately after adjusting the polyelectrolyte solution’s pH value to 3, the solutions were mixed in the order shown in Scheme 1. This led to an overall pH of 3.22 for the mixture. Determined by the intended final KBr concentration in the complex mixture, calculated volumes of pure Milli-Q water and KBr stock solution were first added to a 1.5 mL Eppendorf tube to add up to 0.6 mL. Then, determined by the PDADMA:PAA mixing ratio based upon the moles of repeat units, the calculated volumes of PAA followed by PDADMA were added to the KBr solution. The total polymer concentration was fixed at 0.3 M (to give 0.9 mL), and the KBr concentration was varied from 0–4 M for each mixing ratio studied. The mixing ratio, *f*_PDADMA_, was varied from 0.1–0.9 for which:
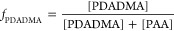
1

After mixing the solutions together, the Eppendorf tubes were vortexed for 30 s using a fixed-speed VWR vortex mixer. For all tests other than UV–vis spectroscopy, the samples were then left to equilibrate for 1 week. After equilibration, the samples were centrifuged using a VWR centrifuge at 1100 *g* for 10 min.

### UV–Vis Spectroscopy

A Hitachi U-4100 UV–vis-NIR spectrophotometer (341-F) was used to measure the turbidity of PDADMA/PAA PECs formed immediately after vortex mixing and after 1 day. A baseline measurement was run against pure Milli-Q water. A wavelength of 750 nm was selected because both pure PDADMA and PAA solutions do not absorb light at this wavelength. The turbidity (*T*) of the mixture was calculated by:
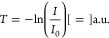
2where *I*_0_ is the incident light intensity of the baseline solution and *I* is the intensity of light passed through the PEC at 750 nm. Turbidity was calculated in absorption units (a.u.)

### Optical Microscopy

A Leica D4M microscope fitted with a Linkam PE120-XY heating and cooling stage was used to image the prepared PDADMA/PAA PECs. Equilibrated and centrifuged samples were decanted and separated into two distinct phases: polymer-rich and polymer-poor phases. A drop of sample from each phase was placed on a glass slide, covered with a coverslip, and placed on the microscope stage. The samples were first imaged at room temperature before the heating cycles were performed. The Linkam stage was heated from 25–75 °C or higher when necessary and cooled back down to 25 °C at 5 °C min^–1^ while taking time-lapse images at 5 s/image. The cooling rate was fixed at 5 °C min^–1^ to match past differential scanning calorimetry (DSC) measurements.^52^ Each image was retaken after 1 day. To avoid concerns of evaporation, the sides of each glass slide and coverslip were sealed with tape and parafilm, and the experimental temperatures observed were kept below 100 °C.

### Thermogravimetric Analysis

Thermal gravimetric analysis (TGA; Q50 TA Instruments) of the coacervate and supernatant for each sample concentration was performed from 25 to 680 °C in a nitrogen environment (60 mL min^–1^). The samples were initially held at a constant temperature of 25 °C for 5 min. The samples were then heated to 110 at 10 °C min^–1^. The samples were then held at 110 °C for 60 min. Subsequently, the samples were then heated to 610 °C at 10 °C min^–1^. The samples were then held at a constant temperature of 610 °C for 90 min. Lastly, the samples were heated to 680 °C at 10 °C min^–1^.

### Variable-Temperature Fourier Transform Infrared (VT-FTIR) Spectroscopy

50-layer pairs of PDADMA/PAA PEMs were prepared on a Germanium (Ge) FTIR crystal using an automated Carl Zeiss HMS slide stainer. First, PDADMA and PAA solutions were prepared at 0.15 M and pH 3 to match the final total concentration of the prepared PECs at a 1:1 mixing ratio. The Ge substrate was cleaned with acetone before deposition. The first layer was deposited by dipping the Ge substrate in PDADMA solution for 15 min, followed by three separate rinse steps for 2, 1, and 1 min in Milli-Q water at pH 3. The second layer was deposited by dipping in the PAA solution, followed by three similar rinses. These two steps were repeated a total of 50 times to form 50 layer-pairs. The PEMs were dried under ambient conditions overnight before VT-FTIR spectroscopy measurements.

The coated Ge FTIR crystal was fitted in a custom-made sample stage from Harrick Scientific Products Inc. connected to a Bruker Tensor II FTIR spectrometer. First, background spectra of the bare Ge crystal were collected at 10 °C intervals from 27 to 75 °C. FTIR spectra were recorded from 4000 to 600 cm^–1^ at a resolution of 2 cm^–1^ in the attenuated total reflectance (ATR) mode at each studied temperature in triplicates.

### Molecular Dynamics (MD) Simulations

The all-atom molecular dynamics (MD) simulations of PDADMA and PAA complexation were performed with the Gromacs 2022.3 package.^53^ To describe the polyelectrolytes, the OPLS-aa force field^54^ was used, with the extension for the ammonium group.^55^ The explicit TIP3P water model^56^ was employed for water. Both, PDADMA and PAA consisted of 40 repeat units. PDADMA molecules were fully charged, while for PAA, a 25% ionization degree was assumed, as determined via ATR-FTIR experiments. 50 molecules of each PE were randomly placed in a cubic simulation box of initial size (28 nm)^3^, which corresponds to *f*_PDADMA_ = 0.5 and a total polymer concentration of 0.3 M. The configurations for the inserted molecules were generated using the built-in Gromacs tool (*gmx insert-molecules*) with PE chain conformations extracted from the dilute solution. The PEs were solvated by explicit water molecules in the system. After solvation, K^+^ and Br^–^ counterions were added to neutralize the system and set the salt concentrations to two different values, i.e., 0.0 and 2.0 M. Ion addition was done by replacing randomly chosen water molecules. The final resulting water content in the system was 97 and 77 wt %, respectively, for KBr concentrations of 0.0 and 2.0 M.

Each system was energy minimized, and a 0.4 ns *NVT* equilibration was performed. Then, the production run in the *NPT* ensemble was run for 200 ns. In all simulations, the Bussi et al. stochastic velocity rescaling algorithm^57^ was used to control the temperature, while the Parrinello–Rahman algorithm was used for the barostat.^58^ The time constants were 0.1 and 2 ps, respectively. The reference temperature and pressure were 298 K and 1 bar, respectively. The long-range electrostatic interactions were calculated using the PME method.^59^ van der Waals interactions were described using the Lennard–Jones potential with a 1.0 nm cutoff. The LINCS^60^ algorithm was used to constrain the bonds between hydrogens and heavy atoms in the PE molecules, while for water molecules the SETTLE^61^ algorithm was used. A 2 fs time step within the leapfrog integration scheme was applied, and the trajectories were written every 1 ps. VMD software was used for visualizations.^62^

## Results and Discussion

### Phase Identification

#### UV–Vis Spectroscopy

PECs were prepared for varying PDADMA fractions (*f*_PDADMA_ = 0.1, 0.3, 0.5, 0.7, and 0.9) and KBr concentrations, as presented in the Experimental Section. For example, *f*_PDADMA_ = 0.5 indicates a 1:1 stoichiometric mixture. The KBr concentration was varied by 0.5 M increments between 0 and 4.0 M; however, at lower concentrations (between 0 and 0.5 M), smaller increments were added to capture the precipitate/coacervate boundary. Due to a solubility of KBr in water at 25 °C of 678 g/L (or 5.7 M),^63^ the highest studied KBr concentration in this study was chosen as 4.0 M.

The prepared complexes were visually inspected after equilibration to obtain an initial phase map (Figure S1). At lower KBr concentrations, two types of precipitate phases were observed: a solid white clump of precipitate and fine precipitate particles dispersed as a milky solution that later settled to the bottom of the vial. At higher KBr concentrations, a coacervate phase was observed as a clear, dense liquid phase at the bottom of the vial.

**Figure 1:**
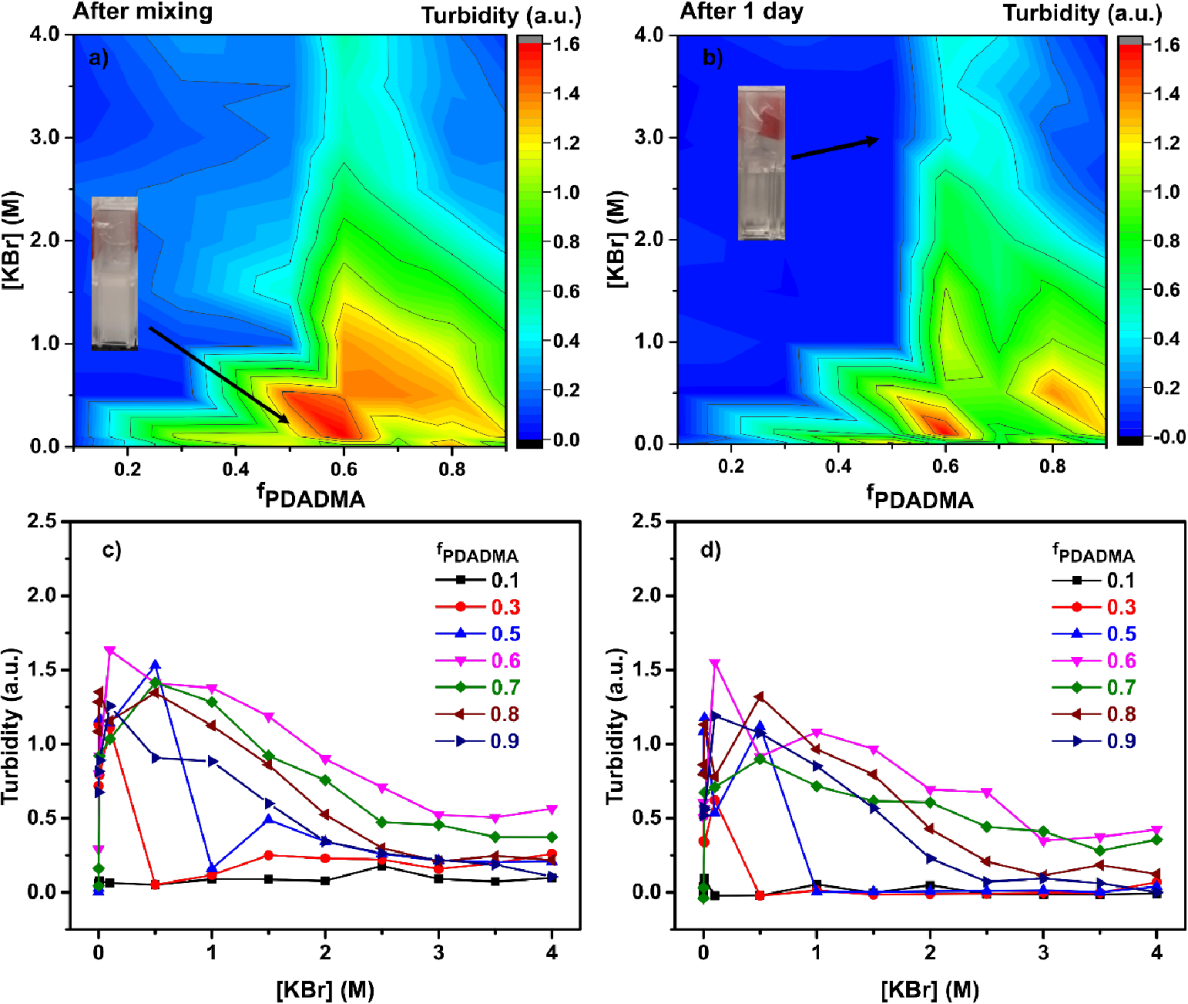
Contour plots showing the turbidity of PDADMA/PAA complex mixtures at room temperature: (a) after mixing and (b) after 1 day without disturbance. Turbidity of PDADMA/PAA complex mixtures as a function of KBr concentration for PECs prepared from different fractions of PDADMA, *f*_PDADMA_: (c) after mixing and (d) after 1 day without disturbance.

Similar to previous studies,^5,14,64,65^ turbidity measurements were used here as a preliminary method of phase identification just after mixing (Figure 1a,c) and 1 day after (Figure 1b,d). Following eq 2, clearer solutions will exhibit higher light transmission and lower turbidity. Turbidity contour maps in Figure 1a,b show an overall reduction in turbidity as the complexes macrophase separate, causing the mixture to become less turbid as the polymer-rich phase settles to the bottom. Higher turbidity was observed for samples prepared with excess PDADMA. This points to the existence of solid precipitates, corroborating earlier reports (but with NaCl as the added salt).^28^ Also, as the KBr concentration increased, the turbidity decreased. This suggests a transition from larger solid precipitates to a coacervate phase, as salt is commonly known to induce phase transitions from solid precipitates to coacervates.^14,25,66,67^Figure 1c,d show more clearly the influence of the KBr concentration on turbidity.

It is noteworthy that the most turbid PEC mixtures occurred when PDADMA was in excess (*f*_PDADMA_ > 0.5). We had expected that more PAA chains would be required to fully compensate the PDADMA chains present due to PAA’s low ionization at pH 3.22, which would have led to more complex formation for excess PAA, but we observed the opposite. This is explained by PAA’s lower linear charge density, PAA’s shift in p*K*_a_ upon complexation, and hydrophobic/hydrogen-bonding interactions among the polyelectrolytes. In its uncomplexed state, the degree of ionization, α, of a cast PAA film was found to be ∼5% but α increased to ∼25% for a dry PDADMA/PAA multilayer film.^44^ Similarly, other studies show the relatively low charge density of PAA and the changes in the charge density and p*K*_a_ with complexation, pH, and ionic strength.^7,43,44,68−70^ Specifically, shifts in the p*K*_a_ of PAA from 6.5 (in solution) to 2.7–4.0 have been reported in PDADMA/PAA complexes and multilayers,^27,41,43,44,68,70−72^ which indicates that PAA is more ionized in the complex than in solution. To verify this, we constructed PDADMA/PAA multilayers and determined that PAA ionization was 27% using ATR-FTIR spectroscopy (Figure S2). Hydrophobic/hydrogen-bonding interactions can also support association, for which studies have shown that complexation at room temperature can occur with PAA at low pH even when predominantly unionized.^73,74^

Taken together, the turbidity measurements indicate that turbidity is highest at low salt concentrations and with excess PDADMA. However, turbidity measurements for phase identification can be limited by the homogeneity of the mixture or its tendency to phase separate and sediment,^64^ as evidenced in comparisons of Figure 1a,b. Therefore, a supporting method is needed for the phase identification.

#### Optical Microscopy

**Figure 2:**
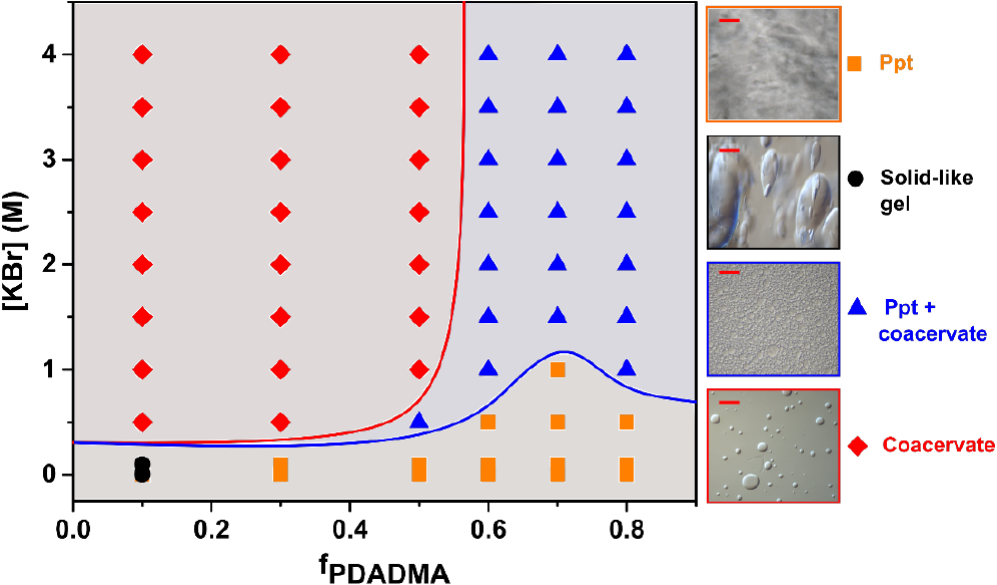
Phase map of PDADMA/PAA complexes at pH 3.22 and 22–27 °C as a function of KBr concentration and PDADMA molar mixing ratio. At the right, optical micrographs showing representative images of precipitate (ppt, orange square), solid-like gel (black circle), ppt with coacervate (blue triangle), and coacervate (red diamond) phases are presented. The scale bar is 50 μm.

After equilibration and centrifugation, the mixtures were imaged by using optical microscopy to identify the phase behavior. Figure 2 presents a phase map for different mixing ratios and KBr concentrations and representative optical micrographs (Figure S3). Images for complexes prepared at 0.9 mole fraction PDADMA (*f*_PDADMA_ = 0.9) could not be obtained because very little of the polymer-rich phase was produced after centrifugation. This is understandable because as *f*_PDADMA_ approaches 1, complexation also approaches the single solution phase. Solid precipitate, coacervate, and two kinds of intermediary phases were identified: a solid-like gel phase at low KBr concentration and a mixed phase of both coacervate and precipitate at high KBr concentration. Precipitates were comprised of large white clumps, finely dispersed white precipitate particles, and an intermediary translucent gel phase. Similar findings of the coexisting precipitate and coacervate phase have been reported for PDADMA/PAA complexes at pH 10 at room temperature.^28,45^ In that study, the basic pH of 10 led to precipitates for PECs with excess PDADMA (*f*_PDADMA_ > 0.625), mixed phases for *f*_PDADMA_ approaching 0.5, and coacervates for PECs with excess PAA (*f*_PDADMA_ < 0.476).^28^ Here, for the acidic pH of 3, we observed similar phase transitions with varying mixing ratios. The presence of the mixed phases makes it challenging to clearly define the coacervate/precipitate boundary. To best describe the boundary in Figure 2, the blue line indicates the onset of coacervation, while the red line indicates a complete transition to the coacervate phase.

Generally, coacervates appeared for KBr concentrations higher than 0.5 to 1 M. However, as the PDADMA content increases, the solid precipitate phase became more prominent and formed a mixed coacervate and precipitate phase. Even at high KBr concentrations (up to 4.0 M) and for excess PDADMA, the solid precipitate phase persisted, coexisting with coacervate. The solution phase was not observed, indicating that the critical salt concentration (CSC) of PDADMA/PAA complexes at pH 3.22 was greater than 4.0 M KBr. Prior work indicates that the CSC can increase substantially for PDADMA/PAA complexes at low pH due to hydrophobic and hydrogen bonding interactions. The relationship for the Flory–Huggins interaction parameter, χ, for PAA and PDADMA at low pH has been used to explain the high CSCs observed in PDADMA/PAA complexes.^7^ Jha et al. predicted that the CSC was greater than 3 M KCl with theoretical models that accounted for solvent interactions for PDADMA/PAA PECs. Elsewhere, the CSC increased beyond experimentally measurable conditions as the pH decreased for PDADMA/PAA in NaCl.^43^ Also, for PDADMA/PAA complexes in KCl, the CSC rapidly increased from 0.5 M at pH 6 to 3 M at pH 4.^27^ Altogether, these past observations agree well with our results for PDAMDA/PAA complexes in KBr at pH 3.22 and room temperature.

The energetics of complexation may also influence phase behavior, in which a shift to a more exothermic complexation enthalpy increases the Gibbs free energy of complexation, thus increasing the driving force for association.^75,76^ We speculate that highly negative Gibbs energies may favor the formation of precipitates in which rapid association may cause the formation of kinetically trapped complexes (i.e., precipitates). For example, isothermal titration calorimetry (ITC) studies have demonstrated that complexation at high pH is endothermic, whereas at low pH, complexation becomes exothermic.^43,77^ As a function of mixing ratio, the enthalpy of complexation was endothermic in the presence of excess PAA but became exothermic in the presence of excess PDADMA.^43^ Similarly, our results show that the presence of excess PDADMA leads to the formation of a precipitate phase.

### Effects of Temperature

#### Optical Microscopy

**Figure 3:**
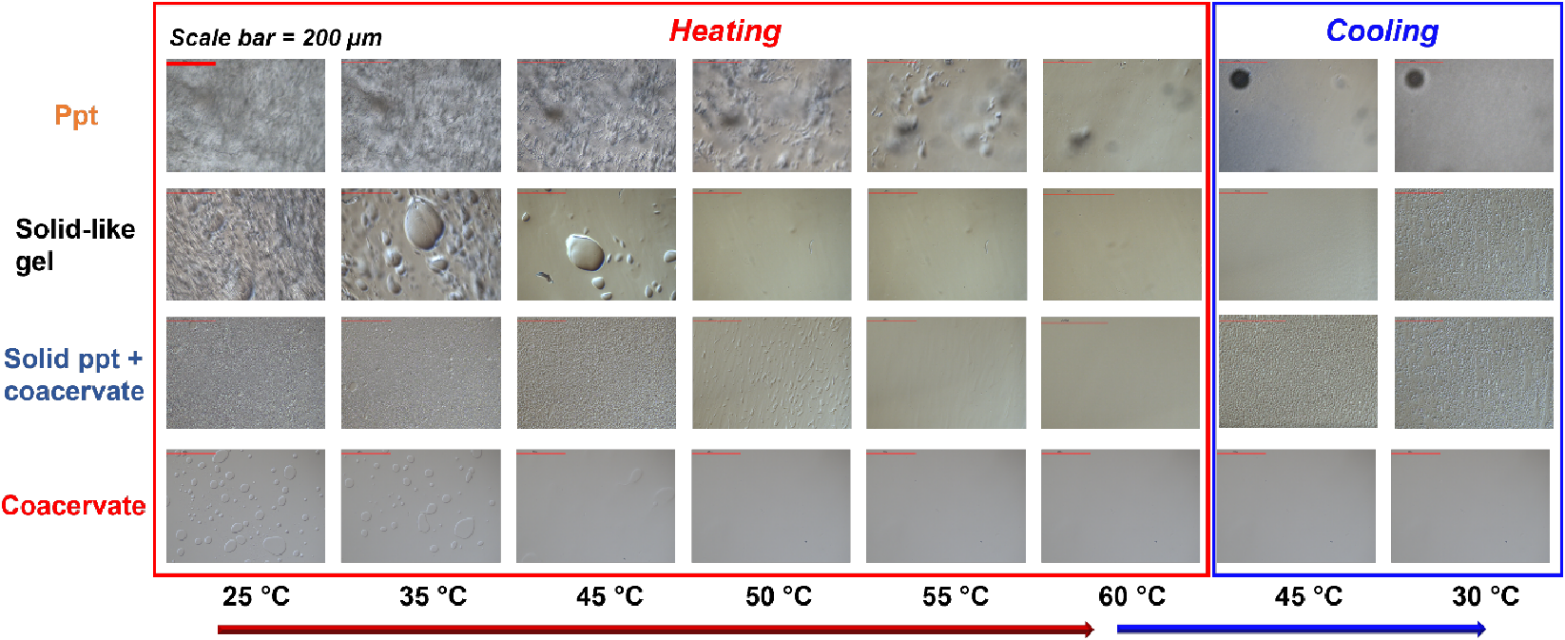
Optical micrographs showing phase transitions with varying temperatures. The top row shows the representative behavior for a precipitate (prepared at *f*_PDADMA_ = 0.6,and [KBr] = 0.001 M). The two middle rows show representative behavior for solid-like gel (*f*_PDADMA_ = 0.3, [KBr] = 0.1 M) and solid precipitate + coacervate (*f*_PDADMA_ = 0.7, [KBr] = 3.5 M) phases. The bottom row shows representative behavior for a coacervate (*f*_PDADMA_ = 0.3,and [KBr] = 3.0 M). The scale bar represents 200 μm. For each representative case, the sample was heated to 60 °C and cooled to 30 °C.

To examine changes in the phase behavior with respect to temperature, the PECs were heated and cooled using a hot stage integrated with an optical microscope. In Figure 3, the top row shows white, opaque precipitates at room temperature that melted away across different layers during heating, leading to a reduced opacity. Upon cooling, the precipitates began to reappear, however, at a smaller particle size (Video S1). The second row shows an intermediary solid-like gel, which at room temperature, was sticky and translucent; upon heating and subsequent cooling, similar to the precipitates, the features disappeared and reappeared. It was, however, difficult to determine if they returned to the gel or precipitate phase distinctly (Video S2). The third row shows a mixed precipitate and coacervate phase, which had features of solid precipitates dispersed within coacervate droplets; upon heating, the coacervate droplets disappeared first, and then the precipitates melted away. Upon cooling, solid-like gel features appeared (Video S3). In general, the solid-containing samples regained some of their features of phase separation upon cooling. The bottom row shows coacervates that at room temperature appeared as droplets of a polymer subphase in a water subphase; upon heating, the droplets coalesced and eventually formed a single solution phase. Upon subsequent cooling, the single solution phase often persisted (Video S4). We found that the reappearance of coacervate droplets may be dependent on the initial concentration of droplets, where droplets were more likely to reappear after heating and cooling when there was a large initial volume fraction of coacervate droplets.

Taken together, the PDADMA/PAA PECs exhibit UCST-type behavior for the entire range of salt and mixing ratios explored. UCST-type behavior has also been reported for PDADMA/PSS coacervates in KBr,^25^ in which a two-phase coacervate state transitions to a single-phase solution upon heating above the UCST.^25,78^

**Figure 4:**
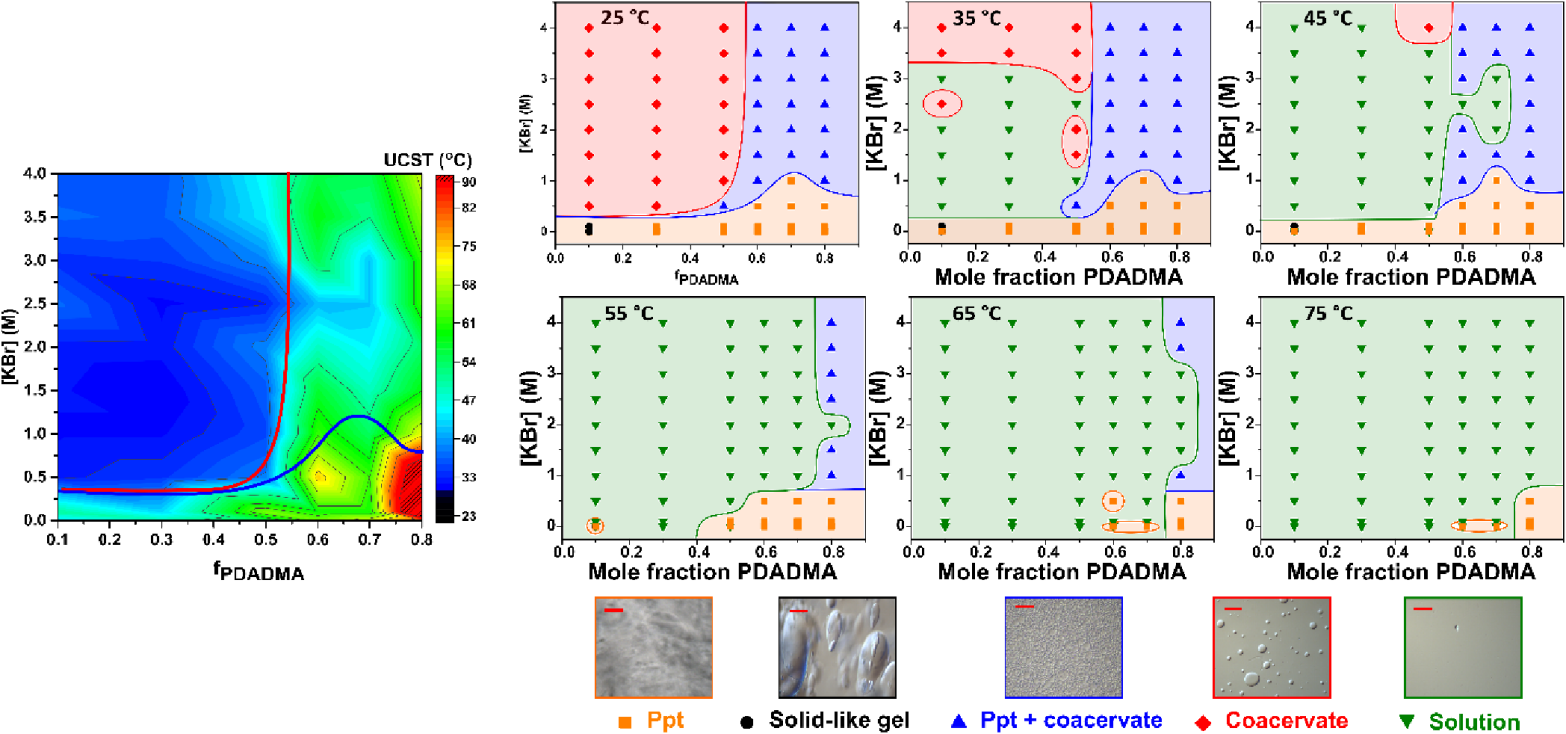
Left) UCST contour plot of PDADMA/PAA complexes at pH 3.22 with varying polyelectrolyte mixing ratios and KBr concentrations as obtained from optical microscopy. The solid red and blue lines demarcate the solid, mixed, and coacervate phases observed initially at room temperature (taken from Figure 2). Right) the effect of temperature on the phase map of PDADMA/PAA complexes at pH 3.22. At bottom, optical micrographs showing representative images of precipitate (ppt, orange square), solid-like gel (black circle), ppt with coacervate (blue triangle), coacervate (red diamond), and solution (green triangle) phases are presented. The scale bar represents 50 μm.

Figure 4a shows the UCST at which all evidence of phase separation ceases to exist. For the coacervate phase, the transition temperature was recorded as the temperature at which the last droplet disappeared. For the mixed phases, the transition temperature was recorded as the temperature at which the coacervate and precipitate particles melted and merged into a uniform solution phase. Last, for the solid precipitate and gel phases, the transition temperature was recorded as the temperature at which all layers of the solid complex melted away.

Overall, the transition temperature was strongly dependent upon whether the complex was initially a precipitate, mixed phase, or coacervate, with precipitates exhibiting the highest transition temperature and coacervates exhibiting the lowest. To illustrate this, we superimposed the phase boundary lines for PDADMA/PAA complexes at room temperature from Figure 2 onto the UCST contour map (Figure 4). For coacervates (above the red line), the UCST ranged from 26 to 40 °C with an average of 34 °C. For solid precipitate-containing complexes (all else below the red line), the UCST ranged from 35 to 87 °C with an average of 53 °C. In general, the UCST increased with *f*_PDADMA_ for a fixed KBr concentration. At *f*_PDADMA_ = 0.8 and low KBr concentrations from 0–0.5 M, the solid precipitates persisted even up to 100 °C, as indicated by the red region. Each heating cycle was capped at 100 °C to avoid evaporation, which prevented exploration at higher temperatures. Figure S4 replots the transition temperatures from the contour map for better visualization.

From temperature-dependent optical microscopy imaging of the complexes, phase maps were constructed for the heating of PDADMA/PAA complexes from 30 to 75 °C, Figure 4b. As the temperature increased, the coacervates were first to undergo a UCST transition at around 35 °C; between 45 and 50 °C the last of the coacervate phase (around *f*_PDADMA_ = 0.5 and 4 M KBr) disappeared. At temperatures above 50 °C, the solid precipitates and solid-like gels in the excess PAA (*f*_PDADMA_ < 0.5) and low [KBr] regions disappeared; at even higher temperatures (∼65 °C), the precipitates with excess PDADMA (*f*_PDADMA_ > 0.5) finally dissolved into the solution phase. Although we do not observe a specific trend between the UCST and ionic strength for PDADMA/PAA coacervates here, Ali et al.^11^ observed a trend between the LCST or cloud point of PDADMA/PSS coacervates and KBr concentration.

In this study, only the UCST-type phase behavior was observed. UCST-type phase transitions are enthalpy-driven, as captured in the temperature dependence of the Flory–Huggins parameter (χ, ),^9,79^ where Δ*H*_m_ is the enthalpy of mixing, *k* is Boltzmann’s constant, *T* is the temperature, *N*_s_ is the number of molecules, and φ_s_ is the volume fraction of solvent. As a result, the UCST can be influenced by changes in the initial polymer concentration, molecular weight, and solvent quality with varying temperature.^9,10,78^ Thermodynamics shows that at the UCST, the Gibbs free energy of the mixture, Δ*G*_m_, equals zero, eq 3. Therefore, for an enthalpy-driven system with Δ*H*_m_ > 0, a UCST can occur with an increase in temperature as χ decreases. At temperatures below the UCST, Δ*G*_m_ > 0 and phase separation occurs; at temperatures above the UCST, Δ*G*_m_ < 0 and no phase separation occurs.
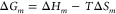
3where Δ*S*_m_ is the entropy of mixing.

For comparison, the UCST behavior has been reported elsewhere for PDADMA/PAA complexes (at pH < 2) using light scattering^10^ to obtain the second virial coefficient, A_2_. For example, DLS results showed a reversible decrease in the hydrodynamic radius of PDADMA/PAA from 69 to 12 nm as the temperature increased from 25 to 60 °C. The authors also examined PDADMA and PAA homopolymers, observing that PDADMA remained soluble from 12 to 60 °C, but PAA was soluble only at temperatures greater than 15 °C (for a concentration of 0.2 M). The insolubility of PAA at low temperatures was attributed to the increased formation of PAA–PAA hydrogen bonds. This UCST-type behavior for PAA has also been identified in a poly(acrylic acid-*co*-acrylonitrile) copolymer due to PAA–PAA hydrogen bonding.^80^ Taken together, these studies demonstrate that the hydrogen bonding contributes to UCST behavior in PAA-containing complexes and copolymers.

We next discuss these results in the context of a recent theory that considers dielectric constant and polymer–solvent interactions. Adhikari et al.^9^ presented several cases of mixed temperature dependence for the dielectric constant and solvent–polymer interaction parameter in polycation-polyanion mixtures. For our system, we know that the dielectric constant of the solvent decreases significantly with temperature. Specifically, the dielectric constant of water decreases from 87.7 at 0 °C to 55.7 at 100 °C,^81^ and for 1 M NaCl aqueous solution, the dielectric constant decreases from 75 at 0 °C to 50 at 100 °C.^82^ Neglecting any polymer–solvent interactions, an increase in the temperature would result in LCST behavior. However, because we consistently observe UCST behavior, we must conclude that polymer–solvent interactions and other interactions (PAA–PAA hydrogen bonding) are key contributors. In comparison, Adhikari et al. discuss that increasing the polymer–solvent interaction parameter (combined with a scaling of χ ∼ T^–1^) can result in the emergence of UCST behavior.^9^ We speculate that polymer–solvent interactions become significant at higher temperatures because PAA–PAA hydrogen bonds can break and, as a result, newly available PAA COOH groups can interact with the solvent.

Chen and Wang^4^ provide an update to the polyelectrolyte complexation theory by considering the entropic contribution from reorganization of the solvent structure, an ‘electrostatic entropy.’ They show that the solvent itself can have a strong influence on the thermodynamics of complexation, especially for weak complexes, such as those explored here. It is possible that the reorganization of the solvent structure, particularly with regard to hydrogen bonding of water with PAA, could contribute to complexation or disassembly for our PDADMA-PAA complexes.

##### ATR-FTIR Spectroscopy

We next explored whether PAA itself contributes to the observation of a UCST in the polycation-polyanion mixture. For example, Litmanovich et al.^10^ showed that the temperature dependence of hydrogen bonding within homopolymer PAA in acidic medium can lead to a thermal phase transition. An indirect method to examine hydrogen bonding is to examine the FTIR spectra of PAA. We examined the temperature-dependence of α of PAA in PDADMA/PAA multilayers at pH 3 using variable-temperature FTIR spectroscopy in ATR mode. Multilayers were used as mimics of the polyelectrolyte complex system. Figure S2a shows the resulting FTIR spectra at temperatures from 27–75 °C in 10 °C intervals. There was a slight decrease in the COOH peak absorbance from 27 to 75 °C, which suggests a reduction in the hydrogen bonding between the PAA carboxylic acid groups. This supports the concept of a hydrogen bonding-driven UCST. Also, for the studied temperature range, α decreased slightly from 27.1% at 27 °C to 26.4% at 75 °C. Therefore, we conclude that PAA–PAA hydrogen bonding diminishes with temperature, weakening the stability of the complex, eventually leading to disassembly at the UCST.

As for PAA ionization with salt concentration, examining the literature tells us that the p*K*_a_ of PAA (and thus its ionization) in a complex can shift depending on the ionic strength of the assembly.^43^ For a PDADMA/PAA PEC assembled in 0.05 M NaCl, the p*K*_a_ of PAA was about 3; for a PEC assembled in 0.3 M, the p*K*_a_ increased to about 3.5.^43^ This leads us to speculate that the p*K*_a_ of PAA in our own PECs likely increases at 2.0 M salt and, therefore, PAA should become less ionized (<25%). This would increase PAA–PAA hydrogen bonding interactions and further stabilize the complex for the 2.0 M KBr PEC. This could be the reason that the UCST increases at a higher salt concentrations (e.g., Figure 4, left).

### Composition of PDADMA/PAA Coacervates and Precipitates

#### Thermogravimetric Analysis (TGA)

To further understand differences between the coacervate and solid precipitate phases that might explain the UCST behavior, thermogravimetric analysis (TGA) measurements were carried out on both the polymer-rich and supernatant phase of PDADMA/PAA complexes at KBr concentrations from 0–4 M, Figure S5. For convenience, we investigated only one *f*_PDADMA_ value, targeting *f*_PDADMA_ = 0.5, because it exhibited both solid precipitate and coacervate phases.

**Figure 5:**
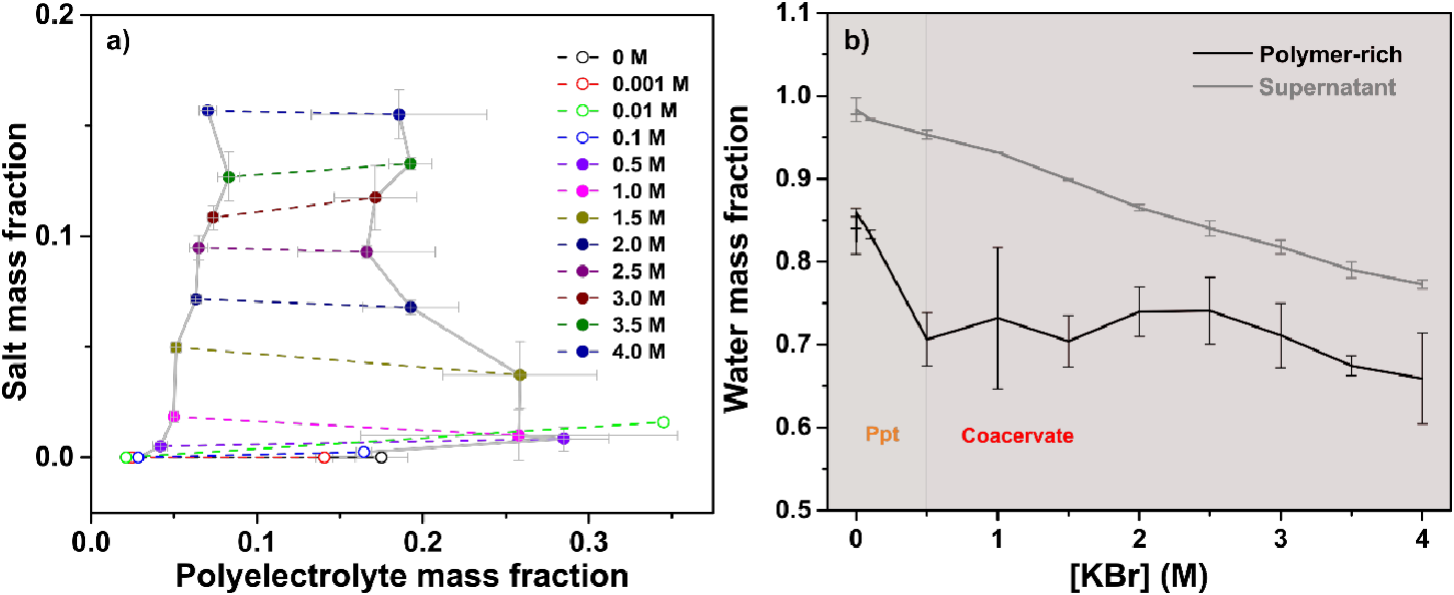
(a) Phase diagram of PDADMA/PAA complexes, including precipitates (open circles) and coacervates (closed circles), at pH 3.22. (b) Water content of PDADMA/PAA complexes at pH 3.22, f_PDADMA_ = 0.5, and varying KBr concentrations obtained from TGA.

Figure 5a shows the resulting phase diagram of a PDADMA/PAA complex at pH 3.22 and *f*_PDADMA_ = 0.5. The boundary on the right represents the composition of the polymer-rich phase, while the boundary on the left represents the composition of the supernatant phase. The closed circles represent the solid precipitate-containing samples ([KBr] = 0–0.1 M), and the closed circles represent the coacervate samples ([KBr] = 0.5–4.0 M). Here, the coacervate/solution boundary does not close at the top to form the expected binodal curve due to the inaccessible critical salt concentration, CSC.^13^ Each dotted line represents a tie line connecting the polymer and salt content of an associated polymer-rich and supernatant phase. The slope of the tie lines of polyelectrolyte complex phase diagrams has been attributed to salt partitioning and thermodynamics.^43,83^ However, due to the large error bars in this study arising from batch–batch differences, it is difficult to draw a strong conclusion on the tie lines’ slopes here.

The phase diagram in Figure 5a allows us to discuss compositional trends of precipitates versus coacervates with salt concentration for *f*_PDADMA_ = 0.5. In the precipitate phase (0 to 0.1 M KBr), a lower polymer content was observed as compared to the coacervate phase. As the salt concentration further increased, coacervates were obtained and the polymer mass fraction increased to a maximum value of 0.28 at 0.5 M KBr. With further increase in the salt concentration, the mass fraction of polymer in the coacervate decreased and then increased again. This looping-in can be attributed to the resulting nonstoichiometric mixing of fully charged PDADMA and partially charged PAA chains. Friedowitz et al. demonstrated this by mixing charged polyacrylamides with pendent ammonium or sulfate groups at varying stoichiometric ratios.^84^ Another explanation for this trend has been described by others as the ‘salting-in’ and ‘salting out’ phenomena of polyelectrolyte solutions.^67,85,86^ Salting-out occurs when the addition of small amounts of salt to a salt-free polyelectrolyte solution leads to precipitation of the polyelectrolyte chains.^86^ Salting-in may occur if the addition of more salt causes the polyelectrolytes to be redissolved.^86^ Salting-in and -out effects can lead to an enclosed binodal phase diagram, observed elsewhere by Li et *al.* for PAH/PAA complexes.^85^

The preceding TGA experiments also allowed for the estimation of the water content in the polymer-rich and supernatant phases, as shown in Figure 5b. As earlier described, an increase in salt concentration breaks down polycation–polyanion ion pairs to form polyelectrolyte–salt ionic pairs. This leads to the transition from solid–liquid phase separation to liquid–liquid phase separation. The water content *w*_water_ decreases steeply from 0.82 at 0 M KBr in the precipitate phase to 0.71 at 0.5 M KBr in the coacervate phase, after which it gradually declines to 0.66 at 4.0 M KBr. Taking the large error bars into consideration, these results point toward an inverse relationship between the ionic strength of complexation and the amount of water in the polymer-rich phase, leading to an increase in the polymer content. While other authors have reported the reverse effect, where increasing the ionic strength leads to an increase in the water content,^13,25^ this dehydration has also been observed and is linked to the salt-stiffening behavior of complexes attributed to osmotic deswelling across the coacervate/precipitate boundary.^67^ The higher water content in the precipitate phase is attributed to the kinetically trapped state of polyelectrolyte chains at low salt concentrations.^50^ This kinetic trapping can also lead to pores within the precipitate phase.^87^ Using molecular dynamics simulations, this effect is further discussed in the next section. For all other mixing ratios, a similar decrease in the water content with increasing KBr concentration was obtained in both the polymer-rich and supernatant phases (Figure S6).

#### Molecular Dynamics (MD) Simulations

For a deeper dive into the phase behavior of PDADMA/PAA complexes, we conducted large-scale, atomistic-detail MD simulations of PE mixtures at KBr concentrations of 0.0 and 2.0 M. To match the experimental conditions as close as possible, the total polymer concentration in the simulations was set to 0.3 M, the PDADMA to PAA ratio was set to 0.5, and the temperature was set to 25 °C. The simulations consisted of 50 PDADMA chains and 50 PAA chains of 40 repeat units each (here called ‘50PDADMA_40_-50PAA_40_’), explicit water molecules, and KBr as explicit solvated ions. Polymer concentration (wt % of polymer) was fixed in the simulations to the mean composition in terms of polymer and salt concentrations used in the experiments, as this gives a sensible mean value for the very small molecular system in the simulations. Notably, in the experiments, the polymer solution phase separates to a polymer-dense and dilute phase.

**Figure 6:**
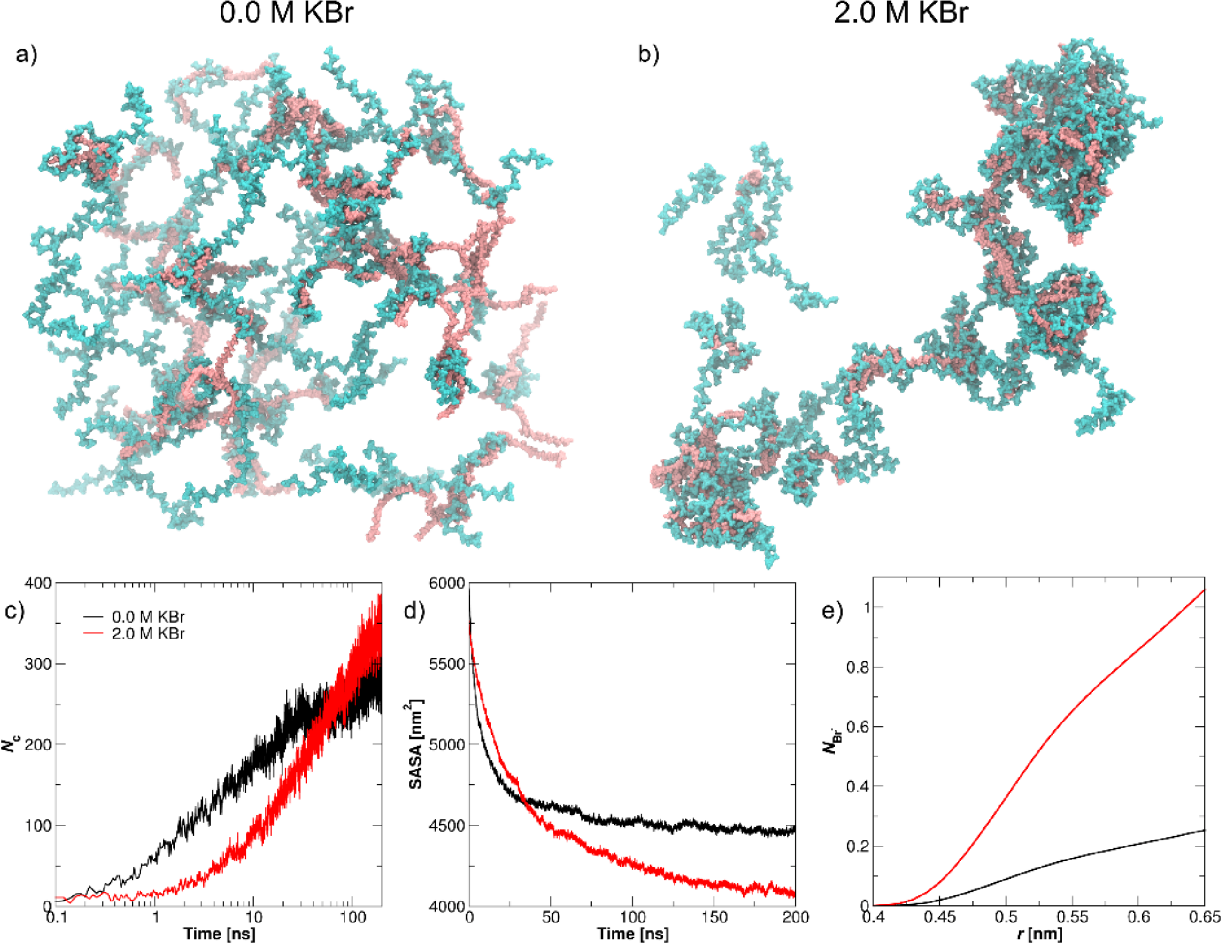
Final configuration of the 50PDADMA_40_-50PAA_40_ systems, simulated for 400 ns using the MD method, at KBr concentrations of a) 0.0 and b) 2.0 M. PDADMA and PAA molecules are highlighted in cyan and pink, respectively. Water and ions were omitted for clarity. c) The number of contacts between N atoms (PDADMA) and O^–^ atoms (PAA) within a distance of 0.5 nm (N_c_) as a function of simulation time. d) Changes in the solvent accessible surface area (SASA) as a function of simulation time. e) The cumulative number of Br^–^ ions around N atoms (N_Br-_) as a function of KBr concentration. The legend in c) applies to d) and e) as well.

The snapshots of the initial configurations are shown in Figure S7, while the final configurations corresponding to a 400 ns simulation duration obtained at both KBr concentrations are presented in Figure 6a,b. The presented visualizations provide qualitative information about the influence of KBr on initial complex formation. At 0 M KBr, relatively small PDADMA/PAA complexes (1PDADMA:1PAA or 1PDADMA:2PAA) are formed (Figure 6a). When a fully charged PDADMA binds with a partially charged PAA (ionization degree of 25% as obtained from ATR-FTIR spectroscopy), the net charge of the resulting 1:1 complex remains highly positive. Therefore, the PDADMA molecules and the formed 1:1 PDADMA/PAA complexes experience strong electrostatic repulsion in the absence of an added salt. This leads to extended configurations of PDADMA, with PAA chains linking the fully charged PDADMA to a networklike, highly porous chain assembly that spreads relatively uniformly into the simulation box. The number of hydrogen bonds between PAA–PAA chains increases from 0.247 ± 0.003 at 0 M KBr to 0.287 ± 0.003 per repeat unit at 2 M KBr. As shown in Figure 6b, increasing the KBr concentration acts to reduce the electrostatic repulsion of the PDADMA chains. This leads to the formation of larger complexes. Additionally, the connectivity of the PE structures in the solution decreases as the PDADMA chains adopt less extended solution conformations, forming more compact, complex structures.

Notably, the 400 ns duration of the all-atom MD simulations allows the characterization of only the initial assembly and its dynamics. Due to the limited time and length scale reach of atomistic detailed MD simulations, the assembled structures should not be considered as representative of the equilibrium but instead provide a comparison of the initial complexation. Equilibrium assembly states may be achieved via coarse-grained,^37^ or mean field and field theory approaches,^29,88^ but at the cost of losing atomic and molecular level resolution, respectively. The significance of the all-atom MD simulations here is that they reveal not only the initial binding response of the PE chains to each other but also the initiation of relaxation via the dynamics and time evolution of the assemblies. Notably, the time scale of the coacervate phase formation is well beyond the all-atom MD simulations, as is the time evolution of the PE assembly to the bulk phase structure.

However, the initial assembly configurations allow for investigating the initial kinetics of PDADMA/PAA complexation at various KBr concentrations. In Figure 6c, we show the time evolution of contacts (<0.5 nm) between the N atom of PDADMA and the O^–^ atom of PAA. At 0 M KBr, the number of contacts increases abruptly to a maximum value, after which further relaxation takes place more slowly than in the system with salt. This suggests that the rapid assembly of PDADMA-PAA PEs at 0 M KBr results in sufficiently strong binding to trap the chains for the duration of the simulation, whereas the excess KBr lubricates chain relaxation. Indirectly, the kinetics changes also point to kinetically trapped states being more likely at 0 M KBr. Also, increasing the salt concentration results in a higher number of close contacts between polyelectrolyte residues. This result indicates that denser complexes should be expected at higher KBr concentrations.

On the other hand, increasing the salt concentration resulted in much slower complexation—about an order of magnitude slower at 2.0 M KBr. Eventually, after ∼80 ns of simulation, the number of contacts between the PE charged groups was higher for 2.0 M KBr than for the salt-free system. Salt addition, therefore, enables further relaxation of the initially formed complexes. This is in line with salt acting as a plasticizer in PECs.^50,89^ The increase in the number of contacts can also be related with the fact that at equimolar mixtures of PDADMA and PAA (*f*_PDADMA_ = 0.5), the charge stoichiometry is around 4:1 due to the PAA being partially charged. The significant excess of positively charged PDADMA residues leads to most of the negatively charged groups from the PAA being neutralized by the PDADMA, even at a lower amount of KBr.

Interestingly, while the number of intrinsic pairs (PDADMA-PAA) is similar, the number of PDADMA-Br^–^ extrinsic pairs increases significantly with the KBr concentration, in contrast to the systems where both PEs are fully charged and the extrinsic and intrinsic sites compete with each other.^90^ This is manifested by the increase in the cumulative number of Br^–^ ions around the N atom of PDADMA, as shown in Figure 6e. It is also worth mentioning that the amount of counterions condensed around PAA will be strongly related to its ionization degree.^91^ In conjunction, the increase in salt concentration results in a larger amount of condensed counterions as well as a decreased average distance between PEs. Both effects are expected to expel water from the complex. This was confirmed in Figure 6d, which shows the solvent accessible surface area (SASA) of polyelectrolyte chains. The significantly lower SASA for 2.0 M KBr suggests that the increase in the salt concentration caused a decrease in the amount of water near polyelectrolyte chains.

Taken together, MD simulations indicate that PDADMA–PAA complexes assembled at 0 M KBr form rapidly, yielding a state with relaxation kinetics significantly slower than those in the presence of excess KBr. The rapid complexation could also lead to entrained water in the complex, which would result in a lower density. As salt concentration increases, complexation is delayed and relaxation kinetics speed up, leading to more ‘equilibrated’ and denser structures with lower water content. In agreement, the experimental results from TGA (Figure 5b) showed that the precipitates, existing at lower KBr concentrations, had a higher water content and were thus less dense than the coacervates at higher KBr concentrations. This difference may explain why solid precipitates exhibited significantly higher UCSTs relative to those of the coacervates. The kinetically trapped precipitates may require more thermal energy to overcome the barrier to disassembly.

As shown in Figure 4, an increase in temperature results in PEC dissolution for almost all investigated salt concentrations and PE molar ratios. In our earlier MD simulations,^52,92^ increasing temperature strongly affects the behavior of water molecules in the PECs. The temperature-mediated increase in the water mobility facilitates solvation of the PE charge groups. This effect is especially pronounced for PDADMA/PAA PECs, where due to the strong affinity of PAA and water, the PDADMA–PAA electrostatic bonding and PAA–water hydrogen bonding are competitive.^92^ Elsewhere, this effect has manifested as significant swelling of PDADMA–PAA multilayers in comparison to e.g., PSS/PDADMA or PSS/PAH PECs.^93^ Therefore, the PDADMA/PAA PEC phase transition observed at elevated temperature (Figure 4) is related to a decrease in the number of PDADMA-PAA intrinsic pairs and to this disruption of PAA–PAA hydrogen bonds, which ultimately leads to PEC dissociation.

## Conclusions

PDADMA/PAA complexes exhibit a rich collection of phase behavior that depends on the temperature, salt concentration, and mixing ratio. Liquid coacervate, solid precipitate, and mixed phases were identified for the complexes prepared at pH 3.22. Low salt concentrations favored the formation of a solid precipitate phase, and high salt concentrations favored coacervate phase formation. In all conditions explored, PDADMA/PAA complexes demonstrated UCST-type behavior. This UCST-type behavior is attributed to the enthalpy-driven thermodynamics of the transition as well as the Flory–Huggins solvent quality contributions, particularly regarding the disruption of the PAA–PAA hydrogen bonds. Solid precipitates demonstrated UCSTs tens of degrees higher than those of liquid coacervates. Solid precipitates contained more water than liquid coacervates. MD simulations showed that under the conditions in which precipitates form, the initial complexation is fast and may cause entrainment of water, resulting in a higher water content in the complex than under the conditions corresponding to the liquid coacervates. Kinetic trapping may explain the precipitates’ significantly higher UCST relative to the coacervates.

In future work, we will explore other pH values of the assembly to modulate the ionization and hydrogen bonding ability of PAA. This may result in different phase and thermal transition behaviors. Future work will also examine how the structures and compositions inferred from the present work might translate into the growth and structure of layer-by-layer assemblies. Taken together, this work has highlighted the phase behavior of complexes at different temperatures, leading to new insights into how solid and liquid-like complexes vary in UCST and structure.

## Supporting Information Available

Phase map from visual identifications, optical micrographs at room temperature and during heating and cooling, ATR-FTIR spectroscopy results, water content of coacervates, and initial configurations used in the MD simulations (PDF)
Videos of UCST behavior observed via optical microscopy (MP4, MP4, MP4, MP4)
