# Supplementary material for: Solid–Liquid–Solution Phases in Poly(diallyldimethylammonium)/Poly(acrylic acid) Polyelectrolyte Complexes at Varying Temperatures
Source: Macromolecules. 2024 Feb 22;57(5):2363–75. doi: 10.1021/acs.macromol.4c00258 (PMC10938883; doi:10.1021/acs.macromol.4c00258)
Supplement: Supplementary file 1 — ma4c00258_si_001.pdf [file ma4c00258_si_001.pdf]

Solid-Liquid-Solution Phases in Poly(diallyldimethylammonium)/poly(acrylic acid) Polyelectrolyte Complexes at Varying Temperatures

Chikaodinaka I. Eneh,[a] *Kevin Nixon,*[a] *Suvesh Manoj Lalwani,*[a] *Maria Sammalkorpi,*[b,c,d] *Piotr Batys,*[e]* *Jodie L. Lutkenhaus*[a,f]*

a. Artie McFerrin Department of Chemical Engineering, Texas A&M University, College Station, Texas 77843, United States

b. Department of Chemistry and Materials Science, Aalto University, P.O. Box 16100, 00076 Aalto, Finland

c. Department of Bioproducts and Biosystems, Aalto University, P.O. Box 16100, 00076 Aalto, Finland

d. Academy of Finland Center of Excellence in Life-Inspired Hybrid Materials (LIBER), Aalto University, P.O. Box 16100, 00076 Aalto, Finland

e. Jerzy Haber Institute of Catalysis and Surface Chemistry, Polish Academy of Sciences, Niezapominajek 8, 30-239 Krakow, Poland

f. Department of Materials Science and Engineering, Texas A&M University, College Station, Texas 77840, USA

TABLE OF CONTENTS

	Page
Figures	S-2
Videos	S-7
References	S-8

FIGURES

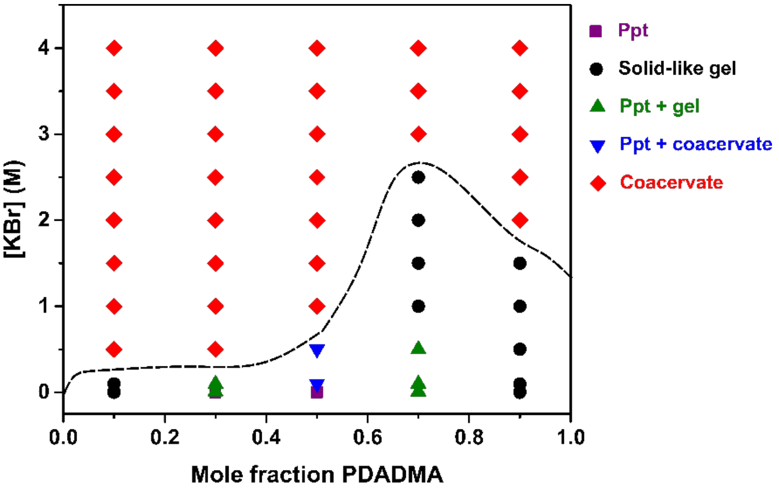

Figure S1. Initial phase map from visual inspection prior to optical microscopy for PECs after seven days of equilibration.

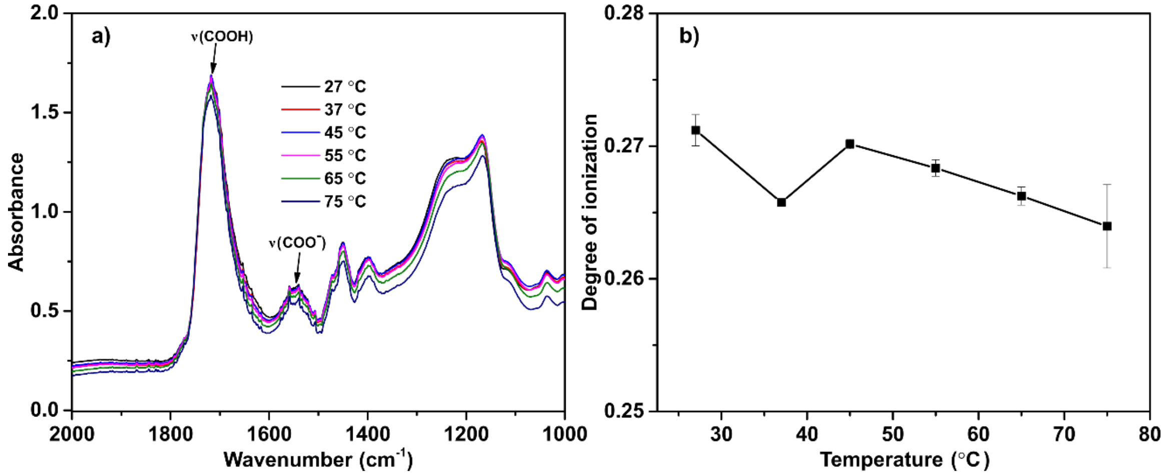

Figure S2 a) ATR-FTIR spectra of 50 layer pairs of PDADMA/PAA multilayers assembled in water at pH 3 and subjected to varying temperatures from 27 – 75 °C. b) Calculated degree of ionization, α. Values of α were calculated using the two absorption bands of the carboxylic acid functional group of PAA: the asymmetric stretch band of COO^- at $\nu = 1540\ cm^{-1}$ and the C=O stretching of un-ionized COOH at $\nu = 1717\ cm^{-1}$. Assuming the same extinction coefficient and using the maximum peak height for both bands, α was calculated using:[1]

degree of ionization, $\alpha = \frac{\nu(COO^-)}{\nu(COO^-)+\nu(COOH)}$.

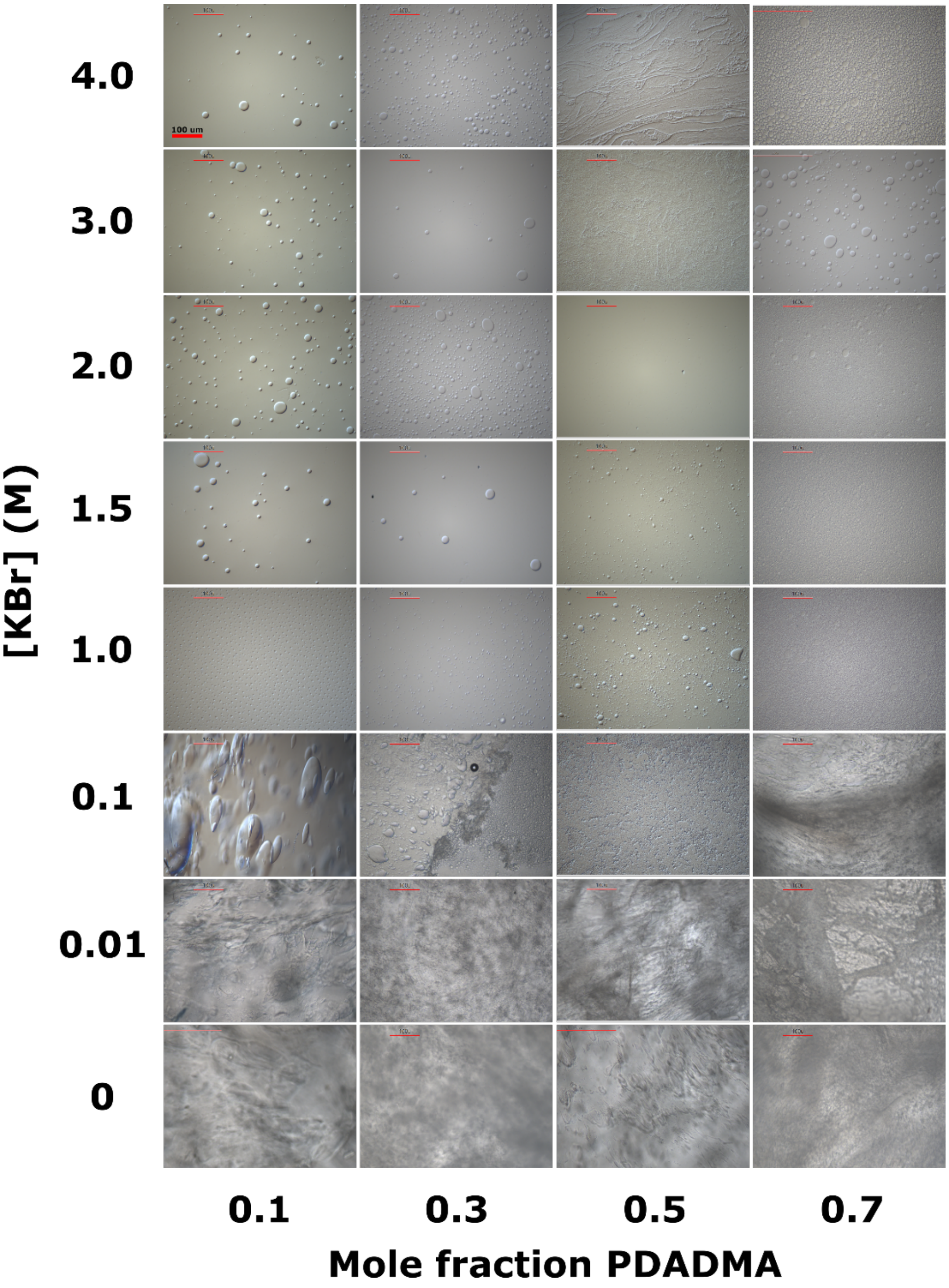

Figure S3. Optical micrographs of all samples used in creating phase map in **Figure 2.**

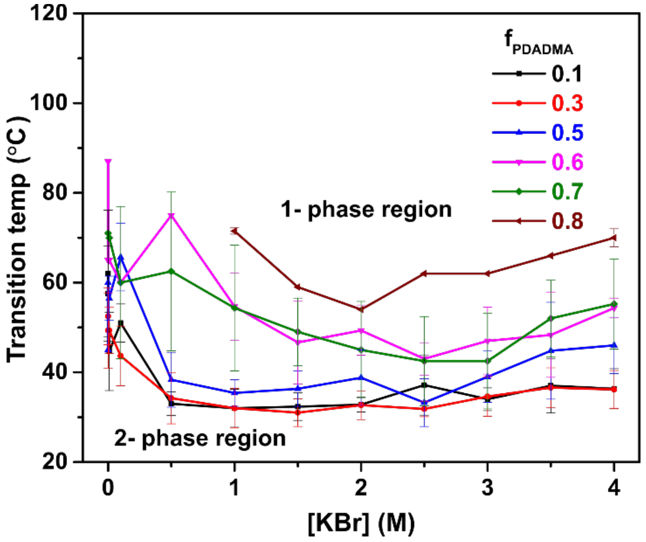

Figure S4. Transition temperature of PDADMA/PAA complexes at pH 3.22 with varying polyelectrolyte mixing ratio and KBr concentration as obtained from optical microscopy.

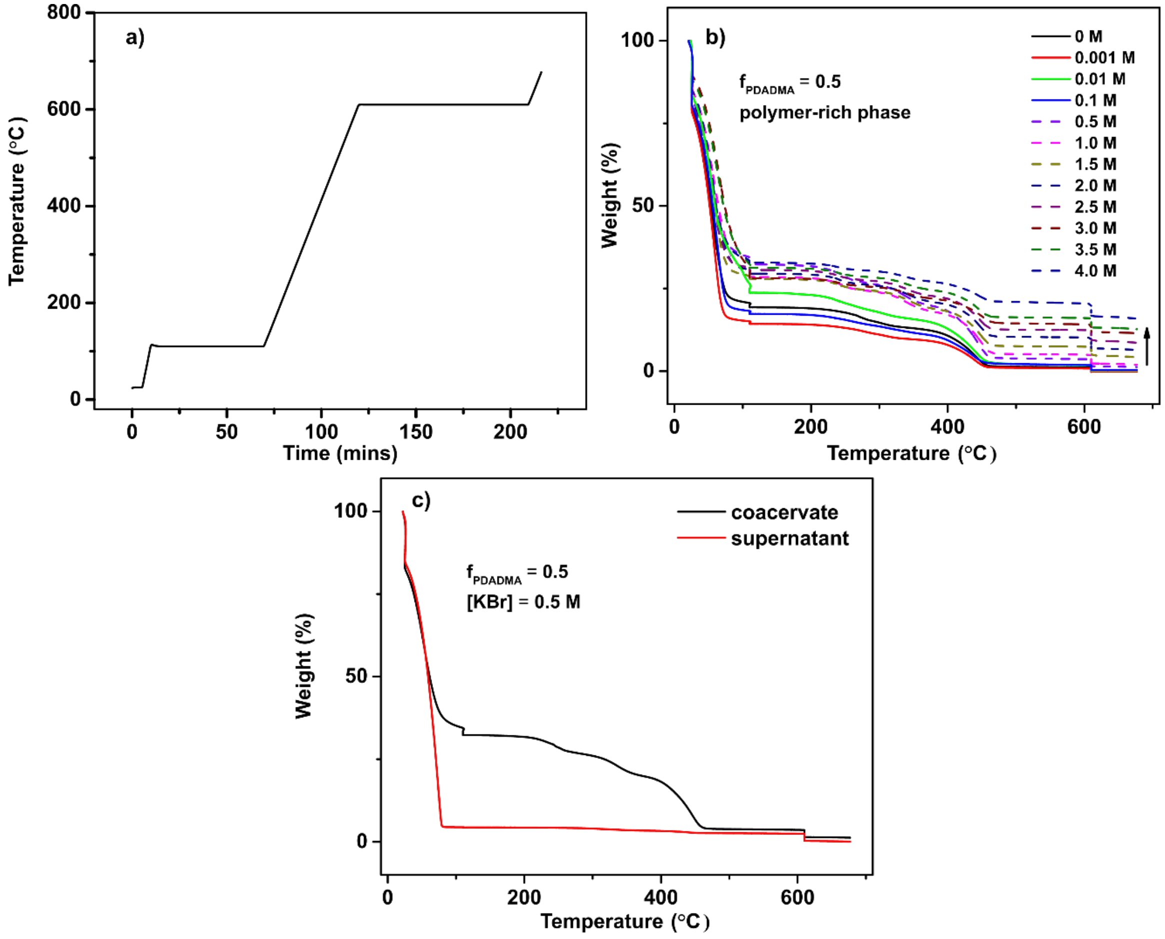

Figure S5. a) Temperature profile of TGA protocol. TGA results for b) polymer-rich phase including precipitate (solid lines) and coacervates (dashed lines) of a PDADMA/PAA complex at f_{PDADMA} = 0.5 at varying KBr concentrations, c) both complex and supernatant phases of PDADMA/PAA complex in 0.5 M KBr. The initial mass loss at 110 °C is ascribed to water, the polymer content was assigned to mass loss from 110 – 610 °C, and the salt content was taken as the remaining mass at 610 °C. As KBr concentration increased, the final weight fraction left behind increased, indicating an increase in the salt content.

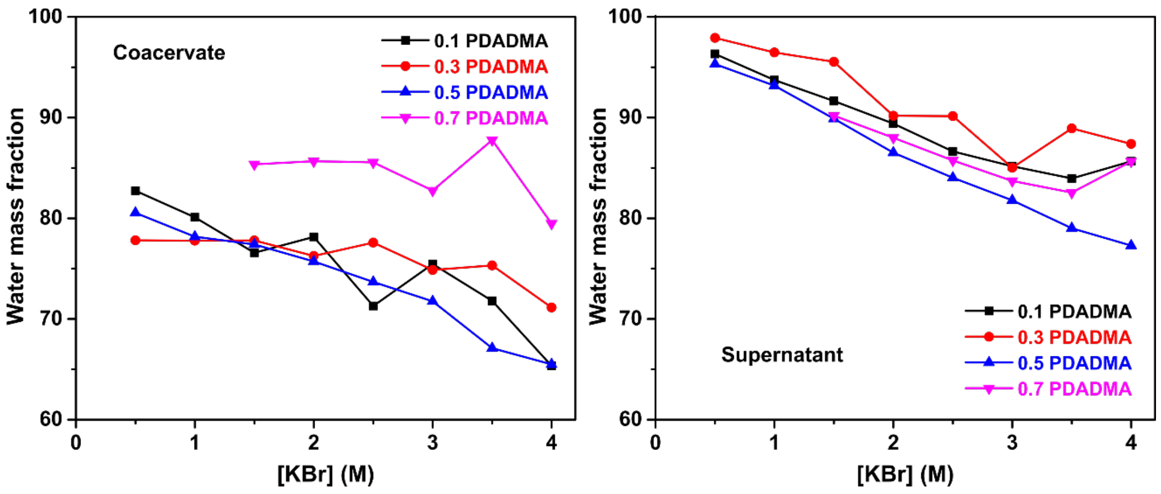

Figure S6. Water content obtained from heating for both coacervate and supernatant phases for all mixing ratios and varying KBr concentration.

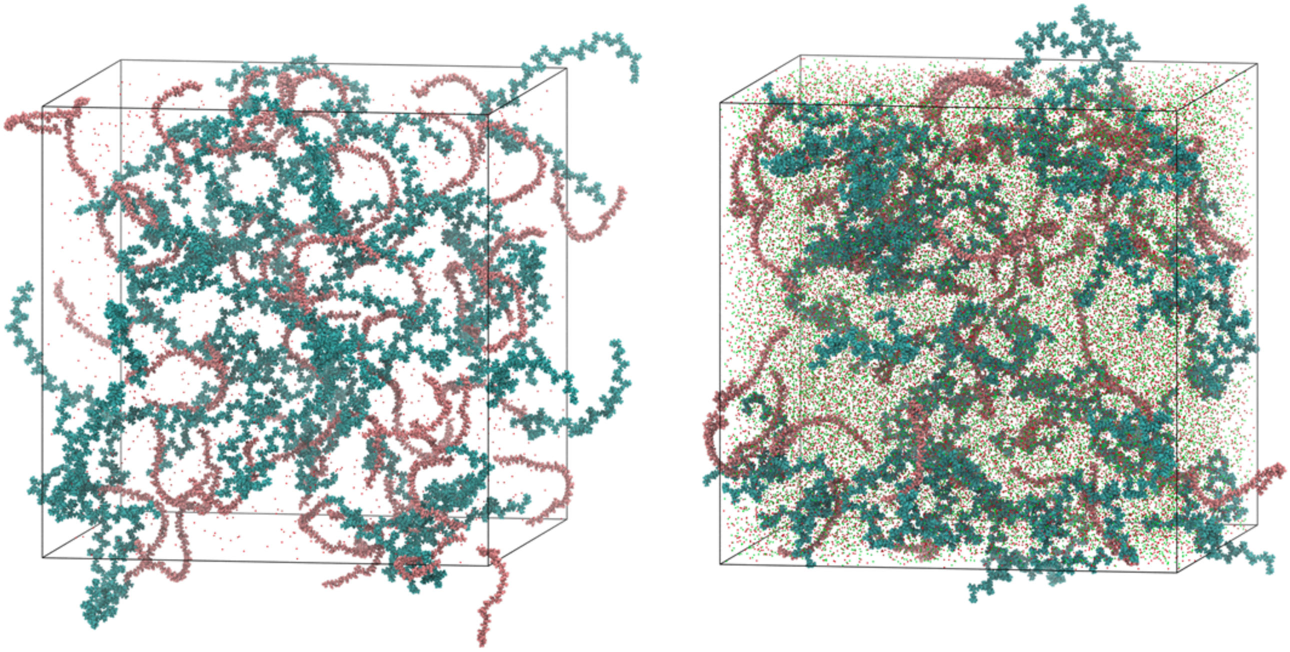

Figure S7. The initial configuration for MD simulations of PDADMA-PAA mixtures at 0 M (on left) and 2 M KBr (on right). The PDADMA, PAA, Br^-, and K^+ are highlighted in cyan, pink, red, and green, respectively. The water molecules are omitted for clarity.

VIDEOS

Videos attached as supporting files.

Video 1. Time-lapse video showing phase transitions during heating and cooling of a sample PDADMA/PAA precipitate

Video 2. Time-lapse video showing phase transitions during heating and cooling of a sample PDADMA/PAA solid-like gel

Video 3. Time-lapse video showing phase transitions during heating and cooling of a sample PDADMA/PAA mixed precipitate and coacervate

Video 4. Time-lapse video showing phase transitions during heating and cooling of a sample PDADMA/PAA coacervate

REFERENCES

1. J. Choi and M. F. Rubner, Influence of the Degree of Ionization on Weak Polyelectrolyte Multilayer Assembly, *Macromolecules*, 2005, **38**, 116-124.
